# Examining the challenges of family recruitment to behavioral intervention trials: factors associated with participation and enrollment in a multi-state colonoscopy intervention trial

**DOI:** 10.1186/1745-6215-14-116

**Published:** 2013-04-30

**Authors:** Rebecca G Simmons, Yuan-Chin Amy Lee, Antoinette M Stroup, Sandra L Edwards, Amy Rogers, Christopher Johnson, Charles L Wiggins, Deirdre A Hill, Rosemary D Cress, Jan Lowery, Scott T Walters, Kory Jasperson, John C Higginbotham, Marc S Williams, Randall W Burt, Marc D Schwartz, Anita Y Kinney

**Affiliations:** 1 Huntsman Cancer Institute, 2000 Circle of Hope, Salt Lake City, UT, 84112, USA; 2 Department of Family and Preventive Medicine, University of Utah, 375 Chipeta Way, Suite A, Salt Lake City, UT, 84108, USA; 3 Utah Cancer Registry, 650 Komas Drive, Suite 106B, Salt Lake City, UT, 84108, USA; 4 Department of Internal Medicine, University of Utah, 30 N 1900 E, Salt Lake City, UT, 84132, USA; 5 Cancer Data Registry of Idaho, 615 N. 7th Street, PO Box 1278, Boise, ID, 83701, USA; 6 New Mexico Tumor Registry, University of New Mexico Cancer Center, MSC 11 6020, 1 University of New Mexico, Albuquerque, NM, 87131, USA; 7 Department of Internal Medicine, Division of Epidemiology and Biostatistics and University of New Mexico Cancer Research and Treatment Center, University of New Mexico School of Medicine, MSC 10 5550, 1 University of New Mexico, Albuquerque, NM, 87131, USA; 8 California Cancer Registry, 1825 Bell Street, Suite 102, Sacramento, CA, 95825, USA; 9 University of Colorado Cancer Center, 13001 E. 17th St., MS F-538, Aurora, CO, 80045, USA; 10 University of North Texas Health Science Center, 3500 Camp Bowie Blvd, EAD 709, Fort Worth, TX, 76107, USA; 11 Institute for Rural Health, University of Alabama, Tuscaloosa Campus, 850 5th Avenue East, Tuscaloosa, AL, 35401, USA; 12 Genomic Medicine Institute, Geisinger Research, Weis Center for Research, 100 N Academy Ave. Mail Stop 26-20, Danville, PA, 17822, USA; 13 Georgetown University, Harris Building, 3300 Whitehaven St., N.W., Washington DC, 20007, USA; 14 Lombardi Comprehensive Cancer Center, 3970 Reservoir Rd NW E501, Washington, DC, 20007, USA

**Keywords:** Cancer, Colorectal, Patient selection, Recruitment

## Abstract

**Background:**

Colonoscopy is one of the most effective methods of cancer prevention and detection, particularly for individuals with familial risk. Recruitment of family members to behavioral intervention trials remains uniquely challenging, owing to the intensive process required to identify and contact them. Recruiting at-risk family members involves contacting the original cancer cases and asking them to provide information about their at-risk relatives, who must then be contacted for study enrollment. Though this recruitment strategy is common in family trials, few studies have compared influences of patient and relative participation to nonparticipation. Furthermore, although use of cancer registries to identify initial cases has increased, to our knowledge no study has examined the relationship between registries and family recruitment outcomes.

**Methods:**

This study assessed predictors of case participation and relative enrollment in a recruitment process that utilized state cancer registries. Participation characteristics were analyzed with separate multivariable logistic regressions in three stages: (1) cancer registry-contacted colorectal cancer (CRC) cases who agreed to study contact; (2) study-contacted CRC cases who provided at-risk relative information; and (3) at-risk relatives contacted for intervention participation.

**Results:**

Cancer registry source was predictive of participation for both CRC cases and relatives, though relative associations (odds ratios) varied across registries. Cases were less likely to participate if they were Hispanic or nonwhite, and were more likely to participate if they were female or younger than 50 at cancer diagnosis. At-risk relatives were more likely to participate if they were from Utah, if another family member was also participating in the study, or if they had previously had a colonoscopy. The number of eligible cases who had to be contacted to enroll one eligible relative varied widely by registry, from 7 to 81.

**Conclusions:**

Family recruitment utilizing cancer registry-identified cancer cases is feasible, but highly dependent on both the strategies and protocols of those who are recruiting and on participant characteristics such as sex, race, or geography. Devising comprehensive recruitment protocols that specifically target those less likely to enroll may help future research meet recruitment goals.

**Trial registration:**

Family Colorectal Cancer Awareness and Risk Education Project NCT01274143.

## Background

Colorectal cancer (CRC) is the second leading cause of cancer mortality in the United States, yet the risk of contracting this lethal disease can vary significantly, owing to genetic factors [1]. A family history of CRC in close relatives increases the lifetime risk of developing the disease by two-to-eight fold [2,3]. Risk varies with the age of onset of CRC in family members, as well as with the number and type of relatives (first-, second-, and third-degree) [4]. Colonoscopy is the recommended screening test for these individuals; however, adherence to screening recommendations is disappointingly low (<50% in most studies) [5-7]. Research on methods to improve colonoscopy screening within this population is crucial to improving the health of at-risk families, yet has been hampered, because of the challenges of recruiting adequate numbers of study participants.

Recruitment of family members to cancer screening behavioral intervention trials poses several unique challenges to researchers. Family history of CRC is not well documented in medical records, which means that researchers must often obtain at-risk relative information by first identifying original cancer cases [8]. These cancer cases are contacted and asked to provide information about potentially at-risk relatives who must then be contacted, screened, and recruited for study participation. At each stage of the process, barriers can affect participation and impair recruitment, which inflates overall study costs and timetables [9,10].

### Recruitment Stage 1: case identification

Cancer registries, particularly state-based cancer registries, represent an ideal source for population-based case identification. However, registries may differ significantly in their research recruitment protocols, which may affect overall recruitment accrual [11]. To date, no research has examined how differences in registry research recruitment polices might affect recruitment of CRC patients. Our study examined initial characteristics of different state registry recruitment protocols and followed registry source as a potential participation and recruitment factor throughout each stage of the recruitment process.

### Recruitment Stages 2 and 3: case participation and relative enrollment

Identifying demographic and medical factors affecting case participation and relative recruitment provides important information to those seeking to optimize participation in future research, yet few studies have reported such factors in cases or relatives recruited to familial cancer behavioral intervention trials. To our knowledge, only three studies have provided predictors of recruitment to familial cancer behavioral intervention trials. A recent melanoma prevention study [12] predicted family participation for relatives who were female, younger at cancer diagnosis, married, and living nearer to the cancer case. Another study, aimed at improving colonoscopy screening, [13] found no predictors of participation in cancer cases who provided at-risk relative contact information. To our knowledge, there is only one published cancer screening behavioral intervention trial that reports factors affecting participation of both cases and siblings [14]. In this study, both male cancer cases and relatives were less likely to participate. The clinical characteristics of cases, including cancer stage, year of diagnosis, and age at diagnosis were assessed but none of these factors was found to significantly influence enrollment [15]. Our study is among the first to report on participation and recruitment characteristics of both cases and different types of relative, such as parents, children, and second-degree relatives. Current clinical guidelines suggest earlier colonoscopy screening for individuals who have two or more second-degree relatives with CRC; thus, examining factors related to enrollment in these individuals is an important aspect of a family intervention trial [6].

This study seeks to build upon previous research of predictors of family-based enrollment in behavioral intervention trials designed to motivate cancer screening. To our knowledge, this is the first multisite family-based CRC study that uses population-based recruitment strategies with multiple state cancer registries to examine baseline demographic and clinical predictors of recruitment of both cancer cases and their first- and second-degree at-risk relatives. Our study is also the first of its kind to report the direct costs associated with family recruitment. Recruitment outcomes of our study may provide useful information to investigators interested in reaching these difficult-to-access populations.

## Methods

### Family CARE project overview

The Family Colorectal Cancer Awareness and Risk Education Project (Family CARE) is a randomized, controlled trial investigating whether a personalized, remote, theoretically-based risk-assessment and telephone counseling intervention is more effective than a mailed, low-intensity targeted print message delivered to individuals who are considered at intermediate familial risk for CRC. At-risk family members who enrolled in the study were randomized by family unit to either the tailored intervention group or the minimal intervention group. A detailed description of the study intervention theory and methods is provided elsewhere [15]. A total of 481 first- and second-degree relatives were enrolled in the study between September 2009 and September 2011. Ethical approval for all study procedures and recruitment protocols were approved by the Institutional Review Boards of participating institutions, which are listed as follows: University of Utah Institutional Review Board; Utah Cancer Registry Advisory Research Committee Resource for Genetic and Epidemiologic Research; Committee for the Protection of Human Subjects through the California Public Health Institute; Idaho Cancer Analysis Work Group; University of New Mexico Human Research Review Committee Institutional Review Board; Colorado Department of Public Health and Environment Institutional Review Board; Intermountain Healthcare Institutional Review Board; Cancer Genetics Network Steering Committee; and University of Colorado Multi-Insitutional Review Board (protocol #10-0243).

#### Participants

Individuals were enrolled in the Family CARE study through a three-stage recruitment process. Eligibility was determined at each stage of recruitment. A CONSORT diagram of the study recruitment process up to enrollment and randomization of relatives is provided in Additional file 1.

#### Recruitment Stage 1: registry case contact

The majority (97.1%) of participants in our study were recruited from five state cancer registries: California, Colorado, Idaho, New Mexico, and Utah. A small number of cases (2.9%) were drawn from Cancer Genetics Network (CGN) registries and hospital-based registries [16]. Registries differed from one another with regard to: (1) whether physician permission or notification was required prior to contacting cases; (2) opt-out and opt-in participation policies; (3) consent process (written, telephone, or passive consent); (4) intensity level of the cases contact and follow-up (defined in Table 1); and (5) cases diagnosis years included in each registry’s sampling frame. Table 1 outlines state-registry-specific protocols.

**Table 1:** State registry case recruitment protocol for the Family CARE Study, 2009 to 2011

**Protocol**	**Case diagnosis years**	**Physician permission type**^**a**^	**Case consent type**	**Target relative risk group**^**b**^	**Contact intensity**^**c**^
California Cancer Registry	2005 to 2008	None	Passive	Group 1	Low
Cancer Data Registry of Idaho	1971 to 2007	Passive	Written only	Group 1, Group 2	High
Colorado Cancer Registry	2003 to 2009	Passive	Written only	Group 1	Moderate
New Mexico Tumor Registry	2000 to 2009	Passive	Written only	Group 1	Low
Utah Cancer Registry	1971 to 2008	None	Phone or written	Group 1, Group 2	High

^a^Passive physician notification provides physicians a time-based opt-out period in which physicians can refuse patient contact for a study.^b^Group 1: one first-degree relative (FDR) of an individual diagnosed with colorectal cancer (CRC) between 40 and 60 years of age. Group 2: one FDR with a relative diagnosed with CRC at any age AND one additional FDR or second-degree relative with a relative diagnosed with CRC at any age (CRC cases must be related to one another).^c^High: both phone and mail follow-up. Moderate: at least one mail follow-up. Low: no follow-up.

CRC cases were eligible to refer their relatives for participation if they met particular criteria for age, cancer site (ICD-O codes: C18.0 to C20.9, excluding 18.1), histology (8140–8143, 8190, 8210, 8211, 8260–8263, 8480, 8481, 8490, 8510), and year of diagnosis, which varied by registry (Table 1). The Utah Cancer Registry and the Cancer Data Registry of Idaho differed from other participating registries in that they received a preselected list of cases that met the family risk criteria for the study from the Utah Population Database, a unique genealogical database containing records for multiple generations of families [17]. This database also allowed study investigators to identify eligible first- and second-degree relatives for possible inclusion in the study.

Once registries had obtained cases consent, information about cancer cases was passed on to Family CARE study staff to begin Stage 2 of the recruitment process.

#### Recruitment Stage 2: cancer case contact by Family CARE staff

To obtain information about potentially eligible relatives, Family CARE staff contacted all registry-identified cases. Cases were mailed an introductory letter, study brochure, and family contact form (FCF), which requested the contact information of potentially eligible relatives.

Cancer cases who did not return the FCF received follow-up calls from Family CARE staff seven days after study packet mailings. As suggested by Durrant *et al*. [18], follow-up calls were made within four time slots (morning, afternoon, evening, weekend) to ensure the highest probability of contact. Staff sent nonresponders a noncontact form, which included options to provide alternate phone numbers, indicate permission to be contacted at a later date, or refuse further participation and contact.

Cases who provided information about their relatives were sent a thank-you letter; and their relatives’ information was grouped by a family identification number, in preparation for Stage 3.

#### Recruitment Stage 3: relative contact by Family CARE staff

Relatives were mailed a consent document, a study brochure, and an initial letter informing them that Family CARE staff would be telephoning them to determine their eligibility and interest in participating, and to answer questions about the study. Relatives were eligible to participate in the study if they had a first-degree relative diagnosed with CRC between the ages of 40 and 49 (participant age, 30 to 74), if they had a first-degree relative diagnosed with CRC between the ages of 50 and 59 (participant age, 40 to 74), or if they had a first-degree relative diagnosed with CRC at age 60 or over, plus an additional first- or second-degree relative diagnosed with CRC at any age (participant age, 40 to 74). Relatives were ineligible for participation if they had a previous cancer diagnosis (other than nonmelanoma skin cancer or a pediatric cancer) or a colonoscopy within the last five years; if they were currently participating in any other research study; if they could not read, write, and speak English, or were mentally incompetent or currently incarcerated; if they had a relative with a molecular or clinical diagnosis of Lynch syndrome or another hereditary cancer syndrome; or if they had received previous clinical or research-based cancer-risk counseling or evaluation or had previously participated in a clinical trial or a behavioral or epidemiologic familial cancer study.

Relatives who were screened and determined to be ineligible received a personalized thank-you letter with information on CRC screening and prevention. If, during the screening process, an ineligible relative disclosed a family history that appeared to indicate a genetic condition (such as Lynch syndrome), the Family CARE staff would make a referral for genetic counseling at a local cancer genetics clinic [19]. Eligible relatives completed a baseline questionnaire by mail, phone, or the Internet. Once baseline questionnaires were completed, relatives were enrolled in the study and randomized to one of the two study arms. Individuals with other participating family members were enrolled into the same study arm, to reduce the risk of cross-contamination.

### Measures

#### Response rate measures

Response rates (RR) for registries and FCF returns were calculated using the RR1 formula for mailed surveys to specifically named persons formulated by the American Association for Public Opinion Research (AAPOR) [20].The RR1 is calculated as the number of complete interviews (I) divided by the number of complete and partial interviews (P) plus the number of noninterviews (refusals (R), noncontacts (NC), others (O)) plus all cases of unknown eligibility (unknown if household occupied (UH), other unknown(UO)):

$$\mathrm{RR}1=I/\left(I+P\right)+\left(R+\mathrm{NC}+O\right)+\left(\mathrm{UH}+\mathrm{UO}\right)$$

For relative enrollment, RRs were calculated using the RR3 formula for mailed surveys to specifically named persons, which accounts for the proportion of individuals with unknown eligibility who actually may have been eligible (*e*):

$$\mathrm{RR}3=I/\left(I+P\right)+\left(R+\mathrm{NC}+O\right)+e\left(\mathrm{UH}+\mathrm{UO}\right)$$

#### Cost measures

We examined direct recruitment costs for cases and relatives at each of the three stages of recruitment. Direct costs included direct staff time and labor for telephone contact, direct staff time and labor to compile and mail all letters and questionnaires, and costs of printing, mailing materials, and postage.

#### Study variable measures

Demographic information for cancer cases (including cancer stage, sex, age, diagnosis age, race, ethnicity, and census tract-level rural or urban residence) was obtained from state cancer registries. Cases provided information on their at-risk relatives including name, age, address, and sex. ZIP codes were used to determine rural or urban status, according to codes established by the United States Department of Agriculture [21]. The relative screening survey included questions about race, ethnicity, personal, and family history of CRC, CRC screening history, and age. Many cases did not provide accurate information about their relatives’ ages, so cross-checking was required to maintain data accuracy.

### Statistical analysis

We generated descriptive statistics to characterize cases and relatives at respective recruitment stages. We then conducted bivariate analysis, calculating crude odds ratios (OR) and 95% confidence intervals (CI), to determine factors associated with participation at each recruitment stage. Differences were tested using Wald chi-square tests. Multivariable logistic regression analyses were performed to generate ORs and 95% CIs of factors that showed association. Fully adjusted models were run to estimate predictors at each stage of study recruitment. Owing to differences in registry recruitment protocols at Stage 1, inter-registry statistical comparisons were not possible. We excluded California from this analysis level because the passive consent recruitment process resulted in only 26 nonconsenting cases; consequently, numbers were too small for predictive analyses. Thus, intra-registry associations for Colorado, Idaho, New Mexico, and Utah are presented. All analyses were conducted using SAS 9.2 (SAS Institute Inc., Cary, NC, 2008).

## Results

### Response response rates

A total of 8,318 individuals were contacted through the five state registries (Table 2). The registry with the highest RR was the California registry (86.9%), which utilized an opt-out protocol wherein individuals uninterested in study contact were required to call the registry to opt out of future contact. The lowest RR was the New Mexico registry (7.7%), which required written consent for future study contact and informed participants about the study through a single mailed information packet.

**Table 2:** Family CARE Study response rates for the three-stage recruitment process of cancer cases and their at-risk relatives, 2009 to 2011.

	**Total selected**	**Ineligible**	**Eligible**	**Consented**	**Did not consent**	**Response rate**
**Stage 1 Registry recruitment**						
California	2,979	226	2,753	2,391	362	86.9%
Colorado	1,133	150	983	335	648	34.1%
Idaho	634	33	601	137	464	22.8%
New Mexico	1,029	3	1,026	79	947	7.7%
Utah	2,543	329	2,214	1,004	1,210	45.3%
Other registry sources^a^	N/A	N/A	245	61	184	24.9%
**Stage 2 Family contact form response**^b^	4,139^c^	256	3,883	2,043	1,840	52.6%
**Stage 3 At-risk relative study enrollment**	2,435	1,394	1,041^d^	481	560	60.4%^e^

^a^Of the other nonstate registry sources utilized in the Family CARE study, only Intermountain Healthcare and the New Mexico Cancer Genetics Network (CGN) contacted cases to obtain consent for Family CARE study contact. Other nonstate registries (Utah CGN, Huntsman Cancer Registry, and Tissue Resource and Applications Core (TRAC)) provided case information directly to Family CARE study staff in accordance with individual registry protocols. Owing to the diverse nature of case ascertainment and consent, it is not possible to identify which of the initially selected cases were counted as ineligible. Thus, these numbers are not included.^b^For the Family Contact Form (FCF), ‘consented’ means that the case returned the FCF, with or without names of eligible relatives, while ‘did not consent’ means that the case either did not return the FCF form or refused to participate. ^c^Individuals who were contacted directly by Family CARE staff (*n* = 132) rather than by participating registries, are included in this number.^d^This number refers to response rate eligibility (differentiated from study eligibility in Additional file 2).^e^Response rates for at-risk relatives were calculated using the AAPOR RR3 formula: The basis for our estimates was calculated from the response rate of relatives known to be eligible.

#### Family contact form response rates

A total of 4,139 FCFs were sent; later, 256 of those who received FCFs were determined to be ineligible. Only 2,043 of the FCFs were returned, giving a return rate of 52.6% (Table 2). Of the FCFs returned, 726 cases listed an average of three (range: 1 to 26 on a single form) potentially eligible relatives for contact.

#### Relative enrollment response rate’s

A total of 2,435 potentially eligible relatives were contacted for the study, with 1,394 determined ineligible during that process (Table 2). There were 364 individuals whose eligibility status was unknown, either through noncontact or because they declined initial participation, whom we had initially categorized as eligible. Using the AAPOR RR3 formula, we calculated the proportion of these individuals likely to be actually eligible as 32.7%, for a total number of 119. Of those estimated to be study eligible (*n* = 628), 481 individuals were randomized into one of the two study arms, resulting in a RR of 60.4%.

#### Cost per participant outcomes

A cost breakdown by recruitment stage by state cancer registry is provided in Table 3. Costs to recruit one state registry case at Stage 1 varied widely, from $5.69 to $28.69 (average cost, $18.15); this spread was largely determined by the intensity of case follow-up. State registries that did not require physician notification, used a single mailing contact, or did not perform follow-up calls (New Mexico and California) had lower registry recruitment costs. Moderate costs occurred in state registries that had a large number of cases and followed moderate-or high-intensity contact regimens. The cost of recruiting at-risk relatives to the study (Stage 3) was slightly higher ($6.14 more) than the cost of contacting CRC cases (Stage 2). These results do not include costs incurred for the 2.9% of cases provided from nonstate registry sites.

**Table 3:** Cost per participant at each of the three stages of case and relative recruitment, 2009 to 2011

	**Cost per participant**^**a**^	**Contact intensity**^**b**^
**Stage 1 Registry recruitment**		
California	$5.94	Low
Colorado	$27.41	Moderate
Idaho	$28.69	High
New Mexico	$5.69	Low
Utah	$23.02	High
**Stage 2 Family contact form case contact cost**	$25.23	High
**Stage 3 At-risk relative recruitment cost**	$31.37	High

^a^Costs per stage only account for direct costs involved in the recruiting process, including direct staff time and labor for telephone contact, direct staff time and labor to compile and mail all letters and questionnaires, and material costs associated with printing, mailing materials, and postage.^b^High: both phone and mail follow-up. Moderate: at least one mail follow-up or one phone follow-up. Low: no follow-up.

#### Predictors of registry consent outcomes

Predictors of participation differed by registry. Adjusted ORs and 95% CIs are presented by state registry in Table 4 (crude ORs by state are presented in Additional file 2). Cases in Utah were less likely to participate if they were older (≥50) when diagnosed with CRC (OR: 0.74, 95% CI: 0.55, 0.98). Nonwhite race was associated with nonparticipation in both Colorado (OR: 0.39, 95% CI: 0.19, 0.76) and Utah registries (OR: 0.21, 95% CI: 0.08, 0.56). Hispanic ethnicity predicted nonparticipation in Utah (OR: 0.39, 95% CI: 0.24, 0.61).

**Table 4:** Predictors of case participation in the Family CARE Study, 2009 to 2011, by state registry.

**Variable**	**Colorado adjusted odds ratio**^**a**^	**Colorado 95% confidence interval**	**Idaho adjusted odds ratio**^**b**^	**Idaho 95% confidence interval**	**New Mexico adjusted odds ratio**^**c**^	**New Mexico 95% confidence interval**	**Utah adjusted odds ratio**	**Utah 95% confidence interval**
**Total number**								
Consent/nonconsent	335/648		137/464		79/947		1,004/1,210	
**Case age group at contact**								
≤49	Referent	Referent	Referent	Referent	N/A	N/A	Referent	Referent
50 to –59	1.29	0.76, 2.18	1.13	0.52, 2.47			1.25	0.87, 1.81
≥60	1.19	0.65, 2.25	0.69	0.27, 1.78			1.14	0.75, 1.75
**Case age group at diagnosis**								
≤49	Referent	Referent	Referent	Referent	Referent	Referent	Referent	Referent
≥50	0.84	0.57, 1.23	1.84	0.98, 3.46	0.88	0.51, 1.52	0.74	0.55, 0.98
**Diagnosis year**^**d**^								
Before 2000			Referent	Referent			Referent	Referent
2000 to 2004	Referent	Referent	2.03	0.79, 5.23	Referent	Referent	1.01	0.81, 1.27
2005 to 2009	0.84	0.57, 1.22	1.71	0.66, 4.41	1.63	0.97, 2.74	1.07	0.85, 1.36
**Cancer stage**								
Local	Referent	Referent	Referent	Referent	Referent	Referent	Referent	Referent
Regional	1.06	0.80, 1.41	1.00	0.67, 1.49	1.44	0.89, 2.32	1.18	0.97, 1.42
Distant	1.15	0.69, 1.91					0.87	0.67, 1.13
**Sex**								
Female	1.19	0.91, 1.57	1.08	0.73, 1.61	1.23	0.76, 1.99	0.89	0.76, 1.06
Male	Referent	Referent	Referent	Referent	Referent	Referent	Referent	Referent
**Race**								
Nonwhite	0.39	0.19, 0.76	0.68	0.14, 3.19	N/A	N/A	0.21	0.08, 0.56
White	Referent	Referent	Referent	Referent			Referent	Referent
**Ethnicity**								
Hispanic	0.89	0.60, 1.32	N/A	N/A	0.83	0.50, 1.37	0.39	0.24, 0.61
Non-Hispanic	Referent	Referent			Referent	Referent	Referent	Referent
**Rural or urban status**								
Rural	0.94	0.63, 1.40	1.18	0.78, 1.78	N/A	N/A	1.06	0.83, 1.36
Urban	Referent	Referent	Referent	Referent			Referent	Referent

^a^Adjusted odds ratio is defined as the full regression model, with all variables included in the analyses. Full individual registry analyses including total numbers and crude odds ratios can be found in Additional file 2. Owingto their passive consent process, California was not included in the analysis as numbers of nonconsenting cases (*n* = 26) were too small to examine.^b^All consenting cases provided by the Idaho registry were of non-Hispanic ethnicity. Thus, ethnicity was excluded from Idaho analyses.^c^Owing to HIPAA privacy concerns, New Mexico did not provide age, race, or rural or urban status of nonconsenting individuals. Thus, these categories are excluded from New Mexico analyses.^d^Different registries provided eligible cases from different diagnosis years based on consideration of total number of eligible cases. Thus, referent for diagnosis year differs by registry.

#### Predictors of FCF return outcomes

Table 5 shows the crude and adjusted ORs for variables associated with FCF return by cases. Both Hispanic ethnicity (OR: 0.62, 95% CI: 0.50, 0.78) and nonwhite race (OR: 0.66, 95% CI: 0.51, 0.84) were negatively associated with FCF return. Rural residents were more likely to return the FCF than those in urban settings (OR: 1.31, 95% CI: 1.08, 1.58). Sex was also significantly associated with FCF return, as women were more likely than men to participate (OR: 1.25, 95% CI: 1.07, 1.46). The strongest association was registry source; individuals recruited from Idaho (OR: 8.28, 95% CI: 5.11, 13.41), Colorado (OR: 7.12, 95% CI: 5.97, 18.98), New Mexico (OR: 3.32, 95% CI: 2.04, 5.41), and Utah (OR: 10.89, 95% CI: 8.08, 14.66) were more likely to participate than individuals recruited from California.

**Table 5:** Predictors of family contact form (FCF) return by cancer cases contacted by the Family CARE Study, 2009 to 2011.

**Variable**	**FCF returned (*n *,%)**	**FCF not returned (*n *,%)**	**Crude odds ratio**	**95% confidence interval**	**Adjusted odds ratio: all variables**	**95% confidence interval**
**Total number**	**2,043 (52.6%)**	**1,840 (47.4%)**				
**Case age group at study contact**						
≤49	199 (47.8%)	217 (52.2%)	Referent	Referent	Referent	Referent
50 to 59	920 (47.4%)	1019 (52.6%)	0.98	0.79, 1.22	1.02	0.76, 1.36
60+	924 (60.5%)	604 (39.5%)	1.67	1.34, 2.08	1.16	0.82, 1.65
**Case age group at diagnosis**						
≤40	60 (69%)	27 (31%)	Referent	Referent	Referent	Referent
41 to 50	600 (49.3%)	618 (50.7%)	0.44	0.27, 0.69	0.82	0.45, 1.48
51+	1,383 (53.6%)	1,195 (46.4%)	0.52	0.33, 0.83	0.84	0.45, 1.57
**Diagnosis year**						
Before 2000	194 (84%)	37 (16%)	Referent	Referent	Referent	Referent
2001 to 2004	406 (85.3%)	70 (14.7%)	1.11	0.72, 1.71	1.30	0.83, 2.05
2005 to 2009	1,443 (45.4%)	1,733 (54.6%)	0.16	0.11, 0.23	1.27	0.80, 2.00
**Ethnicity**^**a**^						
Hispanic	163 (31.2%)	360 (68.8%)	0.39	0.32, 0.47	0.62	0.50, 0.78
Non-Hispanic	1,656 (54%)	1,410 (46%)	Referent	Referent	Referent	Referent
**Race**						
Nonwhite	329 (48.9%)	344 (51.1%)	0.84	0.71, 0.98	0.66	0.51, 0.84
White	1,714 (53.4%)	1,496 (46.6%)	Referent	Referent	Referent	Referent
**Rural or urban status**						
Rural	425 (57.2%)	318 (42.8%)	1.26	1.07, 1.48	1.31	1.08, 1.58
Urban	1,618 (51.5%)	1,522 (48.5%)	Referent	Referent	Referent	Referent
**Sex**						
Female	960 (55.6%)	767 (44.4%)	1.24	1.09, 1.41	1.25	1.07, 1.46
Male	1,083 (50.2%)	1,073 (49.8%)	Referent	Referent	Referent	Referent
**Cancer stage**						
Local	869 (49%)	905 (51%)	Referent	Referent	Referent	Referent
Regional or distant	1,174 (55.7%)	935 (44.3%)	1.31	1.15, 1.48	1.05	0.91, 1.23
**Registry source**						
California	827 (33.9%)	1,611 (66.1%)	Referent	Referent	Referent	Referent
Idaho	115 (83.9%)	22 (16.1%)	10.18	6.40, 16.19	8.28	5.11, 13.41
Colorado	213 (81.9%)	47 (18.1%)	8.83	6.37, 12.24	7.12	5.97, 18.98
New Mexico	51 (64.6%)	28 (35.4%)	3.55	2.22, 5.67	3.32	2.04, 5.41
Utah	837 (86.4%)	132 (13.6%)	12.35	10.09, 15.11	10.89	8.08, 14.66

^a^Not all cases had available ethnicity data.

#### Predictors of relative enrollment outcomes

Table 6 presents the crude and adjusted odds ratios for the association between potential predictive factors and relative enrollment status in the study. Individuals with other family members participating in the study were more likely to enroll than those without family participation (OR: 1.57, 95% CI: 1.06, 2.70). Individuals from Utah were more likely to participate than those recruited from other states (OR: 1.74, 95% CI: 1.17, 2.60). Individuals who had previously undergone a colonoscopy were more likely to enroll than those who had never had a colonoscopy (OR: 1.69, 95% CI: 1.05, 2.70).

**Table 6:** Predictors of family contact form (FCF) return by cancer cases contacted by the Family CARE Study, 2009 to 2011.

**Variable**	**FCF returned (*n *,%)**	**FCF not returned (*n *,%)**	**Crude odds ratio**	**95% confidence interval**	**Adjusted odds ratio: all variables**	**95% confidence interval**
**Total number**	**2,043 (52.6%)**	**1,840 (47.4%)**				
**Case age group at study contact**						
≤49	199 (47.8%)	217 (52.2%)	Referent	Referent	Referent	Referent
50 to 59	920 (47.4%)	1019 (52.6%)	0.98	0.79, 1.22	1.02	0.76, 1.36
60+	924 (60.5%)	604 (39.5%)	1.67	1.34, 2.08	1.16	0.82, 1.65
**Case age group at diagnosis**						
≤40	60 (69%)	27 (31%)	Referent	Referent	Referent	Referent
41 to 50	600 (49.3%)	618 (50.7%)	0.44	0.27, 0.69	0.82	0.45, 1.48
51+	1,383 (53.6%)	1,195 (46.4%)	0.52	0.33, 0.83	0.84	0.45, 1.57
**Diagnosis year**						
Before 2000	194 (84%)	37 (16%)	Referent	Referent	Referent	Referent
2001 to 2004	406 (85.3%)	70 (14.7%)	1.11	0.72, 1.71	1.30	0.83, 2.05
2005 to 2009	1,443 (45.4%)	1,733 (54.6%)	0.16	0.11, 0.23	1.27	0.80, 2.00
**Ethnicity**^**a**^						
Hispanic	163 (31.2%)	360 (68.8%)	0.39	0.32, 0.47	0.62	0.50, 0.78
Non-Hispanic	1,656 (54%)	1,410 (46%)	Referent	Referent	Referent	Referent
**Race**						
Nonwhite	329 (48.9%)	344 (51.1%)	0.84	0.71, 0.98	0.66	0.51, 0.84
White	1,714 (53.4%)	1,496 (46.6%)	Referent	Referent	Referent	Referent
**Rural or urban status**						
Rural	425 (57.2%)	318 (42.8%)	1.26	1.07, 1.48	1.31	1.08, 1.58
Urban	1,618 (51.5%)	1,522 (48.5%)	Referent	Referent	Referent	Referent
**Sex**						
Female	960 (55.6%)	767 (44.4%)	1.24	1.09, 1.41	1.25	1.07, 1.46
Male	1,083 (50.2%)	1,073 (49.8%)	Referent	Referent	Referent	Referent
**Cancer stage**						
Local	869 (49%)	905 (51%)	Referent	Referent	Referent	Referent
Regional or distant	1,174 (55.7%)	935 (44.3%)	1.31	1.15, 1.48	1.05	0.91, 1.23
**Registry source**						
California	827 (33.9%)	1,611 (66.1%)	Referent	Referent	Referent	Referent
Idaho	115 (83.9%)	22 (16.1%)	10.18	6.40, 16.19	8.28	5.11, 13.41
Colorado	213 (81.9%)	47 (18.1%)	8.83	6.37, 12.24	7.12	5.97, 18.98
New Mexico	51 (64.6%)	28 (35.4%)	3.55	2.22, 5.67	3.32	2.04, 5.41
Utah	837 (86.4%)	132 (13.6%)	12.35	10.09, 15.11	10.89	8.08, 14.66

^a^ Due to small numbers (n ≤ 5), those with other family relationships to the cancer case (e.g., nephew, niece) were not included in the analysis.

^b^ Due to limited data availability, rural/urban status for non-consenting cases could not always be determined. Thus, these individuals (n = 161) are missing from the data.

#### Total study participation outcomes

The number of eligible cases who were contacted in order to enroll one eligible relative in the study varied markedly by registry. The largest difference was in the California registry; 81 cases were contacted to enroll one relative. The smallest difference was through the Utah registry, where seven cases were required to enroll one relative. Ratios for all state registries, including Idaho (14:1), Colorado (32:1), and New Mexico (68:1) exhibited inverse dose response levels congruent with the amount of contact intensity that each registry allowed for the initial case contact.

## Discussion

Recruiting at-risk relatives to this behavioral randomized controlled trial was logistically challenging, with large numbers of cancer cases required to enroll one eligible relative. High contact intensity registries (Utah and Idaho) required fewer cases to recruit one eligible relative, whereas the medium contact intensity registry (Colorado) required moderate numbers of cases to enroll one relative and the lowest contact intensity registries (New Mexico and California) required the most cases to recruit one relative.

Although California initially yielded the largest number of cases with the lowest level of effort, individuals contacted through this registry were less likely to participate than individuals contacted using other registries. It is noteworthy that the California registry was the only participating registry that utilized passive consent for study contact, whereby individuals were informed of study contact via a single mailing with no follow-up; and case names were forwarded to Family CARE staff unless they notified the registry that they wished to decline study contact. Other state registries with higher contact intensity (phone follow-up, phone or mail consent required, additional mailings) were found to yield higher participation rates. In other words, the more contact intensity, the more likely cases were to participate. A qualitative study of participant recruitment for familial cancer research by Kreiger *et al*. [22] found that cancer cases approached for familial research expressed concerns about providing information about at-risk relatives for research purposes. It is possible that more intensive cancer registry contact and information about the study alleviated some concerns that cases felt, particularly in registries that performed phone follow-up and could answer specific questions about the study.

Subsequently, both Stages 2 and 3 of the recruitment process required intensive contact measures, including notification calls, mailings, postcards, reminder calls, and second or third mailings. Despite recruitment intensity, initial registry source continued to be predictive of case participation and relative enrollment in our study. Kreiger *et al*. [22] found that individuals were more willing to participate in research if the study were endorsed by a trusted and familiar source. Our results indicate that cases recruited from Utah and Idaho were most likely to return the FCF and that at-risk relatives were more likely to participate if they were Utah residents; this may be result from participants’ familiarity with and recognition of Huntsman Cancer Institute at the University of Utah, the study coordinating site. These findings emphasize the potential impact of participant familiarity with research groups on study participation.

Cancer registry recruitment costs were lower in state registries that utilized low-intensity contact procedures. However, the cases provided by these registries required more intensive and costly recruitment efforts by study staff, and ultimately yielded fewer total eligible relatives than registries with higher contact intensity. For example, the California registry yielded high numbers of potential participants at a relatively low cost, yet engagement of these individuals by study staff proved difficult; and the effect extended into relative recruitment, as only 34 relatives (7% of the total study population) of California cases were recruited into the study. This is an important consideration for future studies, as low initial contact costs may not translate into low total recruitment costs.

Nonwhite race and Hispanic ethnicity were associated with nonparticipation among cancer cases in the Utah and Idaho registries at recruitment Stage 2. This finding is congruent with previous studies that have shown nonwhite race and Hispanic ethnicity to be significant factors of nonparticipation in screening intervention trials [23-25]. Lai *et al*. [26] found that interventions that utilize culturally targeted strategies to recruit underrepresented groups had more effective recruitment outcomes. Our study utilized cancer registries as our recruitment source; more research is needed to determine what, if any, targeted recruitment strategies aimed at increasing racial and ethnic participation would be effective through a cancer registry [27].

In Stage 2, rural cancer cases were more likely to return the form than their urban counterparts. This finding is novel and further investigations are needed to determine what mechanisms influence participation at this level. Our study was designed to reach individuals in geographically underserved settings for remote intervention, which might have appealed to rural cancer cases, making them more likely to participate. This increase in participation by rural cases was not mirrored by an increase in rural relative enrollment, which suggests that recruitment of cases and at-risk relatives might be motivated by different factors. Additional research examining the differences in motivation to participate between cancer cases and their relatives would be valuable, if indeed different recruitment strategies would be more effective in recruiting each population.

More than half of our study participants (52.2%) had another family member enrolled in the study. Family participation was found to be a significant predictor of relative enrollment with individuals more likely to enroll when other family members also participated. This supports the findings of Glanz *et al*. [13], who reported that 63% of participants in their intervention study examining CRC-risk counseling had at least one other first-degree relative enrolled. Familial support has been found to be an important component of adherence to colorectal screening recommendations; our outcomes build on the idea that such support may also influence relative decision to enroll in CRC screening trials [28].

Our intervention targeted both those who had never undergone colonoscopy and those who were overdue for colonoscopy. Individuals were more likely to enroll in our study if they had already undergone colonoscopy before. Known barriers in obtaining a colonoscopy include fear about the procedure and concerns about the preparation; it is possible that individuals who had been through the process had fewer psychological barriers than those who had never experienced the procedure, and were thus more likely to enroll [29]. Future studies with interest in enrolling only participants who have never undergone screening may need to adjust target recruitment goals to reflect these barriers.

One study limitation was our inability to analyze inter-registry predictors of recruitment, owing to differences in registry protocols and in the data provided. Although intra-registry comparisons provide insight into the factors potentially affecting recruitment within a registry, it is impossible to determine whether true similarities or differences exist between all-registry outcomes. This inhibited our ability to determine the true extent to which registry protocols, contact intensity, and diagnosis years influenced the recruitment process.

Another limitation of our study was the restricted amount of information we were able to obtain on nonconsenting cancer cases and on relatives whom study staff were not able to contact directly. Cancer surveillance data collected by registries do not include information concerning education level, income, socioeconomic status, or other potentially influencing factors. Consequently, we could not assess the effect these variables might have on recruitment outcomes. Furthermore, privacy concerns for nonconsenting registry cases restricted the types of data available for analysis. Data limitations were also present in at-risk relatives, as information about eligible relatives was provided by cancer cases; this limited our ability to obtain certain demographic data on nonconsenting relatives.

Although we have provided information about direct costs related to recruitment, a complete cost analysis that accounted for indirect costs, such as institutional fees, administrative salaries, fringe benefits and other items was not feasible within the scope of this paper. However, as our limited data indicate, costs of identification, contact, and recruitment for behavioral interventions depend on many factors. Further research could provide an important perspective on potential ways to minimize recruitment costs while maximizing outcomes in these populations. As indirect costs can vary dramatically by state and institution, including direct cost information might represent more comparable data for design of future trials.

## Conclusions

Our research provides evidence that recruitment of families through cancer registries remains a feasible but time and labor-intensive process, which involves sifting through a significant number of ineligible or uninterested individuals in order to enroll each study participant. Registry source was an important predictor of participation of both cancer cases and their at-risk relatives. Investigators who are interested in conducting other studies using population-based registries might wish to factor the recruitment protocols of an identified cancer registry into time and budgetary considerations, as differing protocols might require more effort and cost in subsequent recruitment stages. Race, ethnicity, sex, and rural residence were predictive of cancer-case participation; while family participation and Utah residency affected relative participation. Future studies that aim to increase population diversity might consider culturally-specific recruitment strategies, or might need to specify oversampling in cancer registries with highly diverse populations. Theoretically guided and culturally targeted recruitment strategies that enhance family support in familial cancer screening interventions might find greater success in enrollment if family units, rather than individuals, are engaged. These findings provide a unique contribution to the literature and emphasize the need for effective recruitment strategies to familial cancer prevention randomized trials that address potential determinants of participation in screening interventions by cancer cases and enrollment of their at-risk family members.

## Abbreviations

AAPOR: American Association for Public Opinion Research; CGN: Cancer Genetics Network; CI: Confidence interval; CONSORT: Consolidated Standards of Reporting Trials; CRC: Colorectal cancer; Family CARE: The Family Colorectal Cancer Awareness and Risk Education Project; FCF: Family contact form; FDR: First-degree relative; OR: Odds Ratio; RR: Response rate; TRAC: Tissue Resource and Applications Core.

## Competing interests

The authors declare that they have no competing interests.

## Authors’ contributions

RGS was involved with data acquisition, paper conception and design, analysis and interpretation of data, and drafted the manuscript. YCAL assisted with conception and design, as well as analysis and interpretation of data and manuscript revisions. AMS was involved with data acquisition, design, interpretation of data, and manuscript revisions. SLE was involved with paper conception and design and data analysis. AR was involved with data acquisition and manuscript revision. CJ, CLW, DAH, RDC, and JL were involved with paper conception, data acquisition, and manuscript revision. STW and KJwere involved with paper conception and manuscript revision. JCH and MSW were involved with paper conception and design and manuscript revision.RWB and MDS were involved with paper conception and design.AYK was involved with data acquisition, paper conception and design, analysis, interpretation of data, manuscript preparation, and manuscript revision. All authors read and approved the final manuscript.

## Supplementary Material

Additional file 1 CONSORT schema of recruitment process for the Family CARE Study, 2009 to 2011. Click here for file

Additional file 2: Table S1 Predictors of recruitment of case from the Colorado State Cancer Registry, 2009 to 2011. **Table S2.** Predictors of case recruitment from the Cancer Data Registry of Idaho, 2009 to 2011. **Table S3.** Predictors of case recruitment from the New Mexico State Cancer Registry, 2010 to 2011. **Table S4.** Predictors of recruitment of cases from the Utah State Cancer Registry, 2009 to 2011. Click here for file
