# Supplementary material for: Examining the challenges of family recruitment to behavioral intervention trials: factors associated with participation and enrollment in a multi-state colonoscopy intervention trial
Source: Trials. 2013 Apr 30;14:116. doi: 10.1186/1745-6215-14-116 (PMC3691526; doi:10.1186/1745-6215-14-116)
Supplement: Additional file 1 — CONSORT schema of recruitment process for the Family CARE Study, 2009 to 2011. [file 1745-6215-14-116-S1.docx]

**Additional file 1, Consort Schema of Recruitment Process for the Family CARE Study, 2009-2011.**

Recruitment Stage 1: Cancer cases contacted (n=8,563)^a^

- Contacted through state cancer registries. (Total Selected = 8, 318)
- Contacted through other registry sources (n=245)

Excluded during registry contact (n=4,556)

- Did not meet inclusion criteria (n= 741)
- Did not consent (n=3,815)

Recruitment Stage 2: Total cancer cases
contacted by Family CARE staff (n=4,139)

- Identified through registries (n=4,007)
- Identified through other sources (n=132)^a^

Excluded during Family Care staff contact (n= 2,096)

- Did not meet inclusion criteria (n= 256)
- Nonresponse (n=1,840)

Recruitment Stage 3: Eligible^b^ relatives contacted by Family CARE staff (n=2,435)

Assessed for eligibility (n=2,071)

Consented eligibility (n=628)

Randomized (n=496)

Not assessed for eligibility (n=364)

- Declined to participate (n=206)
- Cannot contact (n=158)

Excluded during screening (n=1,443)

- Not meeting inclusion criteria (n=1,365)
- Declined to participate (n=78)

Not randomized (n=132)

- Not meeting inclusion criteria (n=14)
- Baseline questionnaire not returned (n=90)
- Refused (n=24)
- Other (n=4)

Randomized but not eligible, excluded (n=15)

Final randomized and enrolled, eligible (n=481)

- 1 FDR, 0 SDR (n=390)
- ≥2 FDR, 0 SDR (n=31)
- 1 FDR, 1 SDR (n= 52)
- 1 FDR, ≥ 2 SDR (n= 6)
- ≥ 2 FDR, 1 SDR (n=2)

FDR: First-degree relative; SDR: Second-degree relative

1. Other non-state registries, (i.e., Utah CGN, Huntsman Cancer Registry and Tissue Resource and Applications Core [TRAC]), passed case information directly to Family CARE study staff in accordance with individual registry protocols. Due to the diverse nature of case ascertainment and consent, it is not possible to identify which cases of the initially selected were counted as “not eligible”. Thus, these numbers are not included in the Stage 1 recruitment outcomes.
2. This consort diagram outlines study eligibility, whereas Table 2 outlines response rate eligibility. Individuals excluded during screening due to refusal, inability to contact, or passive refusal would still be considered eligible when examining response rates, though they would not be considered study eligible. Thus, in determining response rate calculations, individuals would be excluded if they did not meet inclusion criteria (n= 1365 during screening assessment, n= 14 during randomization, n= 15 randomized but later determined not eligible) for a total n= 1041 (2435- 1365- 14- 15 = 1041).
