# Supplementary material for: Examining the challenges of family recruitment to behavioral intervention trials: factors associated with participation and enrollment in a multi-state colonoscopy intervention trial
Source: Trials. 2013 Apr 30;14:116. doi: 10.1186/1745-6215-14-116 (PMC3691526; doi:10.1186/1745-6215-14-116)
Supplement: Additional file 2: Table S1 — Predictors of recruitment of case from the Colorado State Cancer Registry, 2009 to 2011. Table S2. Predictors of case recruitment from the Cancer Data Registry of Idaho, 2009 to 2011. Table S3. Predictors of case recruitment from the New Mexico State Cancer Registry, 2010 to 2011. Table S4. Predictors of recruitment of cases from the Utah State Cancer Registry, 2009 to 2011. [file 1745-6215-14-116-S2.docx]

| **Table S1.** Predictors of Recruitment of Cases from the Colorado State Cancer Registry, 2009-2011. | | | | | | |
| --- | --- | --- | --- | --- | --- | --- |
| **Variable** | **Consent** | **Non-Consent** | **Crude OR** | **95% Confidence Interval** | **Full Model** | **95 % Confidence Interval** |
| **Total Number** | **335** | **648** |  |  |  |  |
| **Case Age Group**  ≤49  50-59  ≥60 | 32 (31.7%)  193 (35.5%)  110 (32.5%) | 69 (68.3%)  351 (64.5%)  228 (67.5%) | Referent  1.19  1.04 | Referent  0.75, 1.87  0.65, 1.68 | Referent  1.29  1.19 | Referent  0.76, 2.18  0.65, 2.25 |
| **Case Age Group at Diagnosis**  ≤49  ≥50 | 102 (36.2%)  233 (33.2%) | 180 (63.8%)  468 (66.8%) | Referent  0.88 | Referent  0.67, 1.17 | Referent  0.84 | Referent  0.57, 1.23 |
| **Diagnosis Year**  2000-2004  2005-2008 | 59 (37.8%)  276 (33.4%) | 97 (62.2%)  551 (66.6%) | Referent  0.82 | Referent  0.58, 1.17 | Referent  0.84 | Referent  0.57, 1.22 |
| **Cancer Stage**  Local  Regional  Distant | 150 (33.1%)  151 (34.5%)  29 (35.4%) | 303 (66.9%)  287 (65.5%)  53 (64.6%) | Referent  1.06  1.11 | Referent  0.81, 1.40  0.68, 1.81 | Referent  1.06  1.15 | Referent  0.80, 1.41  0.69, 1.91 |
| **Sex**  Female  Male | 170 (32.1%)  165 (36.5%) | 287 (63.5%)  165 (36.5%) | 1.22  Referent | 0.94, 1.59  Referent | 1.19  Referent | 0.91, 1.57  Referent |
| **Race**  White  Non-White | 321 (35.4%)  11 (17.5%) | 586 (64.6%)  52 (82.5%) | Referent  0.39 | Referent  0.19, 0.75 | Referent  0.39 | Referent  0.19, 0.76 |
| **Ethnicity**  Hispanic  Non-Hispanic | 44 (32.6%)  291 (34.3%) | 92 (67.4%)  557 (65.7%) | 0.93  Referent | 0.63, 1.36  Referent | 0.89  Referent | 0.60, 1.32  Referent |
| **Rural-Urban Status**  Rural  Urban | 42 (33.1%)  85(66.9%) | 292 (34.2%)  562 (65.8%) | 0.95  Referent | 0.64, 1.41  Referent | 0.94  Referent | 0.63, 1.40  Referent |

OR – Odds Ratio.

| **Table S2.** Predictors of Case Recruitment from the Cancer Data Registry of Idaho, 2009-2011. | | | | | | |
| --- | --- | --- | --- | --- | --- | --- |
| **Variable^a^** | **Consent** | **Non-Consent** | **Crude OR** | **95% Confidence Interval** | **Full Model** | **95 % Confidence Interval** |
| **Total Number** | **137** | **464** |  |  |  |  |
| **Case Age Group**  ≤49  50-59  ≥60 | 17 (18.3%)  73 (27.2%)  47 (19.6%) | 76 (81.7%)  195 (72.8%)  193 (80.4%) | Referent  1.67  1.09 | Referent  0.93, 3.02  0.59, 2.01 | Referent  1.13  0.69 | Referent  0.52, 2.47  0.27, 1.78 |
| **Case Age Group at Diagnosis**  ≤49  ≥50 | 36 (18.5%)  101 (24.9%) | 159 (81.5%)  305 (75.1%) | Referent  1.46 | Referent  0.96, 2.24 | Referent  1.84 | Referent  0.98, 3.46 |
| **Diagnosis Year**  Before 2000  2000-2004  2005-2008 | 6 (12%)  39 (23.5%)  92 (23.9%) | 44 (88%)  127 (76.5%)  293 (76.1%) | Referent  2.25  2.30 | Referent  0.89, 5.68  0.95, 5.57 | Referent  2.03  1.71 | Referent  0.79, 5.23 0.66, 4.41 |
| **Cancer Stage**  Local  Regional/Distant | 62 (23.5%)  75 (22.7%) | 202 (76.5%)  256 (77.3%) | Referent  0.95 | Referent  0.65, 1.40 | Referent  1.00 | Referent  0.67, 1.49 |
| **Sex**  Female  Male | 69 (21.8%)  68 (23.9%) | 247 (78.2%)  217 (76.1%) | 1.12  Referent | 0.77, 1.64  Referent | 1.08  Referent | 0.73, 1.61  Referent |
| **Race**  Non-White  White | 2 (14.4%)  134 (22.9%) | 11 (84.6%)  450 (77.1%) | 0.61  Referent | 0.13, 2.79  Referent | 0.68  Referent | 0.14, 3.19  Referent |
| **Rural-Urban Status**  Rural  Urban | 49 (23.9%)  82 (21.1%) | 156 (76.1%)  306 (78.9%) | 1.17  Referent | 0.78, 1.75  Referent | 1.18  Referent | 0.78, 1.78  Referent |

OR- Odds Ratio
^a^ Ethnicity was not included in Idaho analysis, since all those who consented were reported as Non-Hispanic white.

**Table S3.** Predictors of Case Recruitment from the New Mexico State Cancer Registry, 2010-2011.

| **Variable^a^** | **Consent** | **Non-Consent** | **Crude OR** | **95% Confidence Interval** | **Full Model** | **95 % Confidence Interval** |
| --- | --- | --- | --- | --- | --- | --- |
| **Total Number** | **79** | **947** |  |  |  |  |
| **Case Age Group at Diagnosis**  ≤49  ≥50 | 21 (8.8%)  58 (7.4%) | 219 (91.2%)  728 (92.6%) | Referent  0.83 | Referent  0.49, 1.40 | Referent  0.88 | Referent  0.51, 1.52 |
| **Diagnosis Year**  Before 2000  2000-2004  2005-2008 | N/A 23 (5.3%)  56 (9.4%) | N/A  407 (94.7%)  540 (90.6%) | N/A  Referent  1.84 | N/A  Referent  1.11, 3.03 | N/A  Referent  1.63 | N/A  Referent  0.97, 2.74 |
| **Cancer Stage**  Local  Regional  Distant | 34 (7.34%)  41 (10.4%)  N/A | 429 (92.7%)  353 (89.6%)  N/A | Referent  1.46  N/A | Referent  0.91, 2.36  N/A | Referent  1.44  N/A | Referent  0.89, 2.32  N/A |
| **Sex**  Female  Male | 40 (8.4%)  39 (7.1%) | 438 (91.6%)  509 (92.9%) | 1.19  Referent | 0.75, 1.89  Referent | 1.23  Referent | 0.76, 1.99  Referent |
| **Ethnicity**  Hispanic  Non-Hispanic | 28 (7.4%)  51 (7.9%) | 349 (92.5%)  598 (92.1%) | 0.94  Referent | 0.58, 1.52  Referent | 0.83  Referent | 0.50, 1.37  Referent |

OR – Odds Ratio
^a^ Due to privacy concerns for non-consenting cases, New Mexico did not provide information on case age and rural/urban status. The racial variable was also excluded from analyses because all consenting cases were reported as white.

| **Table S4.** Predictors of Recruitment of Cases from the Utah State Cancer Registry, 2009-2011. | | | | | | |
| --- | --- | --- | --- | --- | --- | --- |
| **Variable** | **Consent** | **Non-Consent** | **Crude OR** | **95% Confidence Interval** | **Adjusted OR** | **95% Confidence Interval** |
| **Total Number** | **1004** | **1210** |  |  |  |  |
| **Case Age Group**  ≤49  50-59  ≥60 | 100 (45.5%)  341 (47.2%)  563 (44.3%) | 120 (54.5%)  382 (52.8%)  7008 (55.7%) | Referent  1.07  0.95 | Referent  0.79, 1.45  0.72, 1.27 | Referent  1.25  1.14 | Referent  0.87, 1.81  0.75, 1.75 |
| **Case Age Group at Diagnosis**  ≤49  ≥50 | 252 (49.2%)  752 (44.2%) | 260 (50.8%)  950 (55.8%) | Referent  0.82 | Referent  0.67, 0.99 | Referent  0.74 | Referent  0.55, 0.98 |
| **Diagnosis Year**  Before 2000  2000-2004  2005-2008 | 238 (45.2%)  387 (45.1%)  379 (45.9%) | 289 (54.8%)  471 (54.9%)  447 (54.1%) | Referent  0.99  1.03 | Referent  0.80, 1.24  0.83, 1.28 | Referent  1.01  1.07 | Referent  0.81, 1.27  0.85, 1.36 |
| **Cancer Stage**  Local  Regional  Distant | 466 (44.5%)  392 (48.4%)  136(40.8%) | 582 (55.5%)  418 (51.6%)  197 (59.2%) | Referent  1.17  0.86 | Referent  0.97, 1.41  0.67, 1.11 | Referent  1.18  0.87 | Referent  0.97, 1.42  0.67, 1.13 |
| **Sex**  Female  Male | 564 (46.5%)  440 (44.1%) | 650 (53.5%)  559 (55.9%) | 0.91  Referent | 0.76, 1.07  Referent | 0.89  Referent | 0.76, 1.06  Referent |
| **Race**  White  Non-White | 998 (45.8%)  5 (15.2%) | 1182 (54.2%)  28 (84.8%) | Referent  0.21 | Referent  0.08, 0.55 | Referent  0.21 | Referent  0.08, 0.56 |
| **Ethnicity**  Hispanic  Non-Hispanic | 27 (26.7%)  973 (46.1%) | 74 (73.3%)  1136 (53.9%) | 0.43  Referent | 0.27, 0.67  Referent | 0.39  Referent | 0.24, 0.61  Referent |
| **Rural-Urban Status**  Rural  Urban | 145 (47.1%)  857 (45.2%) | 163 (52.9%)  1039 (54.8%) | 1.08  Referent | 0.85, 1.37  Referent | 1.06  Referent | 0.83, 1.36  Referent |

OR- Odds Ratio
